# Regulation of Gut Microflora by *Lactobacillus casei* Zhang Attenuates Liver Injury in Mice Caused by Anti-Tuberculosis Drugs

**DOI:** 10.3390/ijms24119444

**Published:** 2023-05-29

**Authors:** Yue Li, Liangjie Zhao, Changyu Sun, Jingyi Yang, Xinyue Zhang, Sheng Dou, Qinglian Hua, Aiguo Ma, Jing Cai

**Affiliations:** 1 School of Public Health, Qingdao University, Qingdao 266021, China; 2020026470@qdu.edu.cn (Y.L.); 2021024467@qdu.edu.cn (L.Z.); 2019213198@qdu.edu.cn (C.S.); 2019213179@qdu.edu.cn (J.Y.); 2020216056@qdu.edu.cn (X.Z.); 2020216062@qdu.edu.cn (S.D.); 2020026482@qdu.edu.cn (Q.H.); magfood@qdu.edu.cn (A.M.); 2 Institute of Nutrition and Health, Qingdao University, Qingdao 266021, China

**Keywords:** anti-tuberculosis drugs, liver injury, gut microflora, *Lactobacillus casei*, TLR4–NF-κB–MyD88 pathway

## Abstract

The gut–liver axis may provide a new perspective for treating anti-tuberculosis drug-induced liver injury (ATDILI). Herein, the protective effect of *Lactobacillus casei* (Lc) was investigated by modulating gut microflora (GM) and the toll like receptor 4 (TLR4)–nuclear factor (NF)-κB–myeloiddifferentiationfactor 88 (MyD88) pathway. C57BL/6J mice were given three levels of Lc intragastrically for 2 h before administering isoniazid and rifampicin for 8 weeks. Blood, liver, and colon tissues, as well as cecal contents, were collected for biochemical and histological examination, as well as Western blot, quantitative real time polymerase chain reaction (qRT-PCR), and 16S rRNA analyses. Lc intervention decreased alkaline phosphatase (ALP), superoxide dismutase (SOD), glutathione (GSH), malondialdehyde (MDA), and tumor necrosis factor (TNF)-α levels (*p* < 0.05), recovered hepatic lobules, and reduced hepatocyte necrosis to alleviate liver injury induced by anti-tuberculosis drugs. Moreover, Lc also increased the abundance of *Lactobacillus* and *Desulfovibrio* and decreased *Bilophila* abundance, while enhancing zona occludens (ZO)-1 and claudin-1 protein expression compared with the model group (*p* < 0.05). Furthermore, Lc pretreatment reduced the lipopolysaccharide (LPS) level and downregulated NF-κB and MyD88 protein expression (*p* < 0.05), thus restraining pathway activation. Spearman correlation analysis indicated that *Lactobacillus* and *Desulfovibrio* were positively correlated with ZO-1 or occludin protein expression and negatively correlated with pathway protein expression. *Desulfovibrio* had significant negative relationships with alanine aminotransferase (ALT) and LPS levels. In contrast, *Bilophila* had negative associations with ZO-1, occludin, and claudin-1 protein expressions and positive correlations with LPS and pathway proteins. The results prove that *Lactobacillus casei* can enhance the intestinal barrier and change the composition of the gut microflora. Moreover, *Lactobacillus casei* may also inhibit TLR4–NF-κB–MyD88 pathway activation and alleviate ATDILI.

## 1. Introduction

Tuberculosis (TB) is one of the most common causes of death from infectious disease in adults worldwide and has been considered a global public health emergency for the past 25 years [1]. As first-line anti-tuberculosis drugs, isoniazid (INH), pyrazinamide, and rifampicin (RFP) have potential hepatotoxicity [2]. Rifampicin is a hepatic enzyme inducer, and isoniazid is an inhibitor of hepatic enzymes. According to the 2016 version of the Roussel Uclaf Causality Assessment Method (RUCAM), isoniazid and rifampicin cause drug-induced liver injury (DILI) in clinical settings [3]. Anti-TB drugs are the prescribed drugs that have most often induced DILI in Eastern countries, comprising 26.6% of cases, according to a meta-analysis of 33,294 patients [4]. The clinical manifestations of anti-TB drug-induced liver injury (ATDILI) are loss of appetite, nausea, vomiting, fatigue, and discomfort or pain in the liver area. In severe cases, liver failure may occur, which may be life-threatening [5]. Physicians commonly prescribe hepatoprotectant capsules such as silibinin to prevent liver injury during anti-TB treatment episodes. However, the protective effect of hepatoprotectants could not be demonstrated in a randomized controlled trial; worse, the trial even found that hepatoprotectants could cause potential liver damage [6]. In addition, silibinin may have poor bioavailability because of its extremely low water solubility and poor intestinal absorption [7]. Moreover, the preventive usage of hepatoprotectans may add extra economic burden to patients, in addition to the controversial safety issue related to the risk of new adverse events induced by hepatoprotectans as drugs are metabolized through the liver [8]. Therefore, a focus on reducing damage to the liver is of great significance for the treatment of tuberculosis.

The occurrence and progression pathogenesis of ATDILI Involve drug metabolism and an inflammatory response; however, many factors remain unknown [9]. In recent years, with the introduction of the concept of the gut–liver axis, some researchers have boldly proposed that liver disease is also related to the gut microflora (GM) [10,11]. When normal liver physiology is destroyed, specific components of bacteria, such as lipopolysaccharide (LPS), will enter the liver from the intestinal cavity through the portal vein, causing excessive inflammation, further damaging the tissues [12], and aggravating the original liver disease [13]. In addition, LPS binds to cell surface receptors, which may regulate the toll like receptor 4 (TLR4)–nuclear factor (NF)-κB signaling pathway, increasing inflammation [14]. Some existing studies have suggested that LPS-induced inflammation can be attenuated by modulating the TLR4–NF-κB–myeloiddifferentiationfactor 88 (MyD88) pathway [15,16,17]. NF-κB is essential in regulating inflammatory signaling pathways and contributes to liver disease [18]. Moreover, TLR4 and MyD88, associated with NF-κB, also play important functions in regulating the TLR4–NF-κB–MyD88 pathway. On the basis of these studies, we speculated that targeting the TLR4–NF-κB–MyD88 axis could represent a potential strategy for mitigating ATDILI.

Probiotics have been used in recent years to improve liver injuries in mice, including high fat diet (HFD)/CCl_4_-induced liver injury and nonalcoholic fatty liver disease [19,20]. For example, *Akkermansia muciniphila* and its derivatives could enhance the intestinal integrity and anti-inflammatory responses of the colon and liver tissues, and subsequently prevent liver injury in HFD/CCl_4_ mice [19]. As a widely studied strain, *Lactobacillus casei* is widely distributed in the mammalian intestines [21]. *Lactobacillus casei* Zhang (LcZ) was shown to have protective effects against various organ injuries [22,23] and antibiotics [24]. Wang et al. found that LcZ could reduce the LPS/d-galactosamine-induced proinflammatory cytokine and hepatic inflammation by modulating the TLR–mitogen-activated protein kinase–peroxisome proliferators-activated receptor γ signaling pathway [23]. Moreover, pretreatment with LcZ protected against LPS/d-galactosamine-induced liver injury in rats and was associated with an inhibition of TLR4 signaling [25]. These results indicate that *Lactobacillus casei* might be beneficial to liver injury. In this paper, we evaluated the protective effect of *Lactobacillus casei* Zhang on anti-tuberculosis drug-induced liver injury in mice and explored whether *Lactobacillus casei* plays a protective role by regulating the gut microflora and TLR4–NF-κB–MyD88 pathway.

## 2. Results

### 2.1. Lactobacillus casei Attenuated Liver Injury Induced by Anti-TB Drugs in Mice

**Figure 1:**
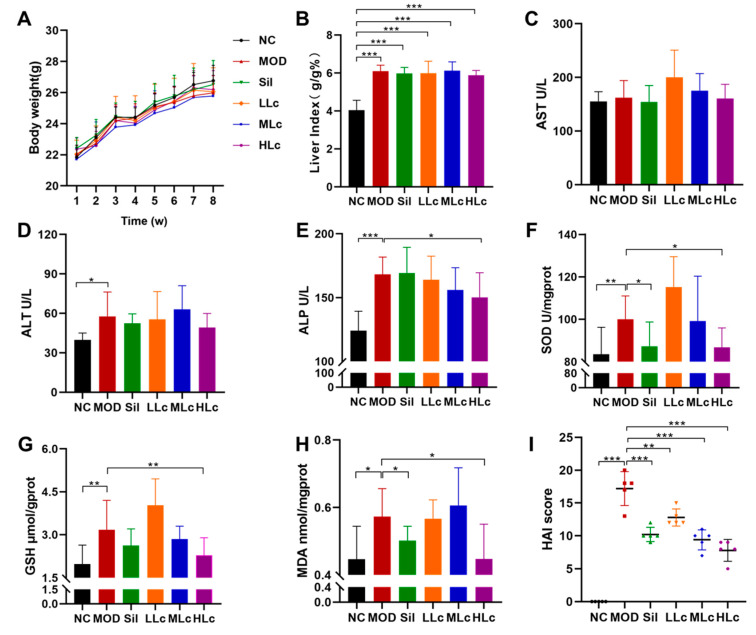
*Lactobacillus casei* attenuated liver injury induced by anti-tuberculosis drugs in mice: (**A**) body weight curve; (**B**) liver index (liver weight/body weight); (**C**) serum aspartate aminotransferase (AST) level; (**D**) serum alanine aminotransferase (ALT) level; (**E**) serum alkaline phosphatase (ALP) level; (**F**) liver superoxide dismutase (SOD) level; (**G**) liver glutathione (GSH) level; (**H**) liver malondialdehyde (MDA) level; (**I**) histological activity index score. NC, normal control group; MOD, model group; Sil, positive control (silibinin) group; LLc, low-dose *Lactobacillus casei* group; MLC, medium-dose *Lactobacillus casei* group; HLC, high-dose *Lactobacillus casei* group. N = 5 for HAI score, N = 9 for others; * *p* < 0.05, ** *p* < 0.01, *** *p* < 0.001.

**Figure 2:**
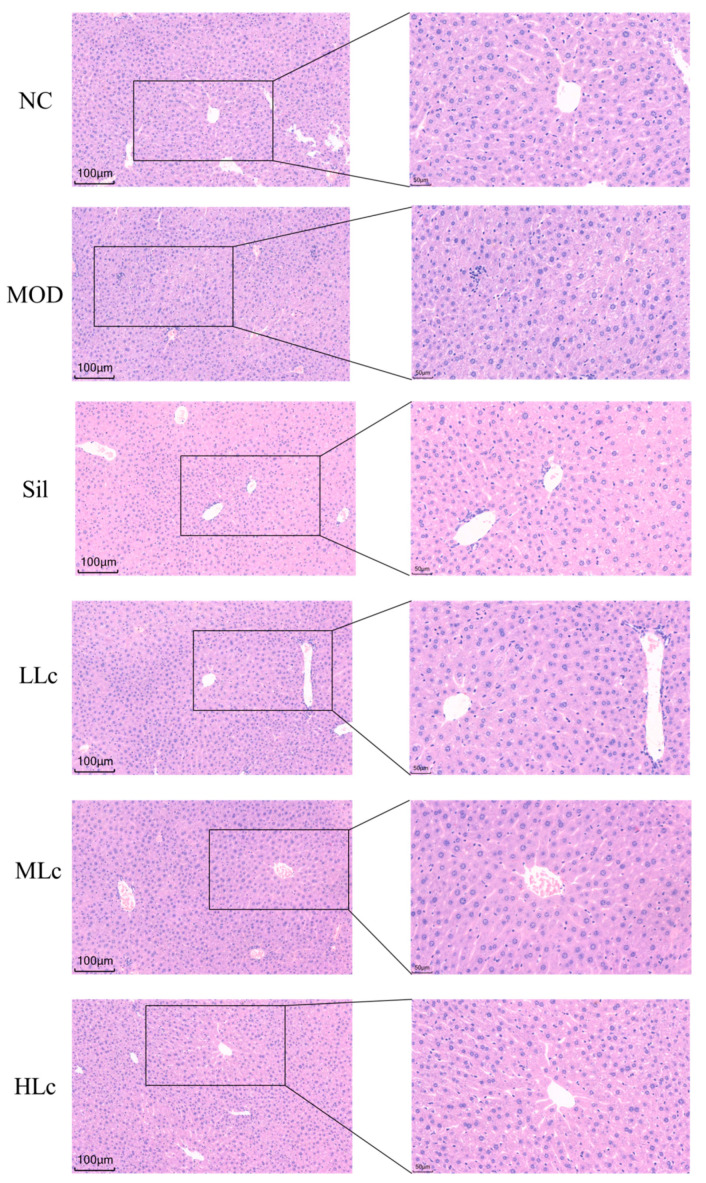
Histological observations of livers (liver hematoxylin and eosin (HE) staining in each group, 200× and 400×). NC, normal control group; MOD, model group; Sil, positive control (silibinin) group; LLc, low-dose *Lactobacillus casei* group; MLC, medium-dose *Lactobacillus casei* group; HLC, high-dose *Lactobacillus casei* group. N = 6.

Figure 1A describes the weekly body weight of mice. The body weight did not differ significantly among the groups. Figure 1B reveals a substantial difference in liver index between the control group and the five anti-TB drug intervention groups (*p* < 0.05). INH and RFP increased ALT and ALP levels in serum compared with normal mice. A high dosage of Lc reversed the elevated ALP levels induced by anti-TB drugs (Figure 1D,E). In addition, as shown in the photomicrographs of liver hematoxylin and eosin (HE) staining (Figure 2), hepatic lobule disappearance, inflammatory cell infiltration, and hepatocellular necrosis were observed in the MOD group. By contrast, silibinin and *Lactobacillus casei* alleviated liver injury in mice, showing the recovery of hepatic lobules and inflammatory cell infiltration, as well as a marked reduction in the number of necrotic hepatocytes. As shown in Figure 1I, the histological activity index (HAI) score of the MOD group was significantly higher than that of the control group; however, after intervention with silibinin or *Lactobacillus casei*, the score was significantly reduced (*p* < 0.01). Moreover, increased superoxide dismutase (SOD), glutathione (GSH), and malondialdehyde (MDA) were found in mice after anti-TB drug exposure. On the contrary, the HLc group had reduced levels of SOD, GSH, and MDA (Figure 1F–H). Altogether, the results demonstrate that *Lactobacillus casei* had a protective effect against liver harm caused by INH and RFP.

### 2.2. Lactobacillus casei Renovated the Intestinal Barrier in Mice with Anti-TB Drugs

**Figure 3:**
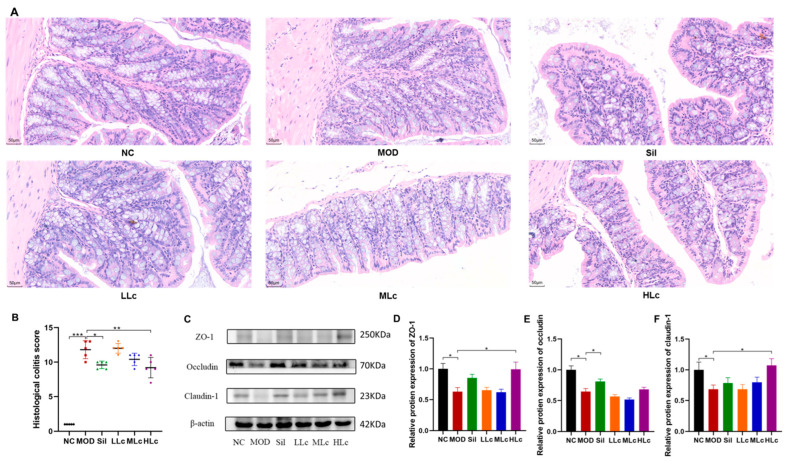
*Lactobacillus casei* renovated the intestinal barrier injured by anti-tuberculosis drugs in mice: (**A**) representative photographs of HE-stained sections of colons (400×); (**B**) histological colitis score; (**C**) protein expression of zona occludens (ZO)-1, occludin, and claudin-1 measured by Western blot; (**D**) quantitative analysis of ZO-1 protein expression; (**E**) quantitative analysis of occludin protein expression; (**F**) quantitative analysis of claudin-1 protein expression. NC, normal control group; MOD, model group; Sil, positive control (silibinin) group; LLc, low-dose *Lactobacillus casei* group; MLC, medium-dose *Lactobacillus casei* group; HLC, high-dose *Lactobacillus casei* group. N = 5 for histological colitis score, N = 6 of HE staining, N = 3 for others; * *p* < 0.05, ** *p* < 0.01, *** *p* < 0.001.

Increased epithelial structural damage and inflammation occurred in the colonic mucosa of anti-TB drug-treated mice compared with control mice. Serious crypt damage also occurred in the model group, which was manifested as a reduced number of crypts and structural disorder. *Lactobacillus casei*-treated animals, on the other hand, had fewer damaged crypts, lower inflammatory cell infiltration, and a thicker epithelial layer (Figure 3A). A histological colitis score was determined for the colon, showing a significant increase with anti-TB drugs and a significant decrease after intervention with silibinin or high-dose Lc (*p* < 0.05) (Figure 3B). The levels of tight junction (TJ) proteins in the MOD group were considerably lower than those in the NC group (*p* < 0.05) (Figure 3C–F). The mice treated with high-dose Lc showed substantial increases in the expression of zona occludens (ZO)-1 and claudin-1 compared to the model group (*p* < 0.05) (Figure 3C,D,F). Therefore, *Lactobacillus casei* could upregulate TJ protein expression and strengthen intestinal barrier function.

### 2.3. Lactobacillus casei Modulated the Diversity of Gut Microbiota in Mice with Anti-TB Drugs

**Figure 4:**
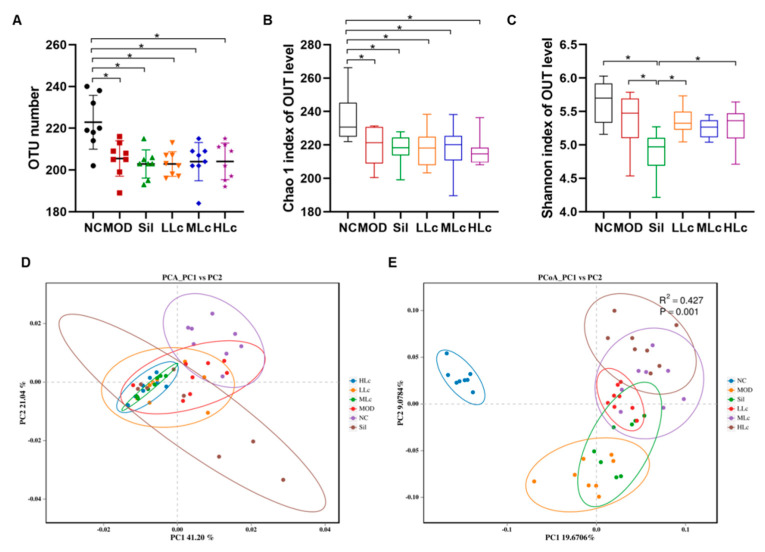
*Lactobacillus casei* modulated diversity of gut microbiota in mice with anti-tuberculosis drugs: (**A**) operational taxonomic units (OTUs); (**B**) Chao 1 index of α diversity; (**C**) Shannon index of α diversity; (**D**) Principal component analysis (PCA) plots; (**E**) principal coordinate analysis (PcoA) based on binary Jaccard at OTU level. Permutational multivariate analysis of variance (PERMANOVA) using the weighted UniFrac similarity coefficient based on binary Jaccard was also performed. NC, normal control group; MOD, model group; Sil, positive control (silibinin) group; LLc, low-dose *Lactobacillus casei* group; MLC, medium-dose *Lactobacillus casei* group; HLC, high-dose *Lactobacillus casei* group. N = 8; * *p* < 0.05.

The average number of operational taxonomic units (OTUs) in other groups was considerably lower than in the NC group (*p* < 0.05) (Figure 4A). Compared to the NC group, the Chao1 index of the anti-TB drug-treated groups decreased significantly (*p* < 0.05) (Figure 4B). In contrast, the Shannon index was similar in all groups except the Sil group (Figure 4C). The β diversity analysis is shown in Figure 4D,E. The findings reveal that the microbial composition of each group exhibited a distinct cluster (Figure 4D,E). According to principal coordinate analysis (PCoA) and permutational multivariate analysis of variance (PERMANOVA), the model groups and the NC group were separate, whereas the three Lc-treated groups shaped partially coincident clusters in the ordination plot compared to the MOD group, suggesting that Lc induced significant modulations in the GM profiles (Figure 4E).

### 2.4. Lactobacillus casei Modulated Microbial Taxonomic Profiles in Mice with Anti-TB Drugs

**Figure 5:**
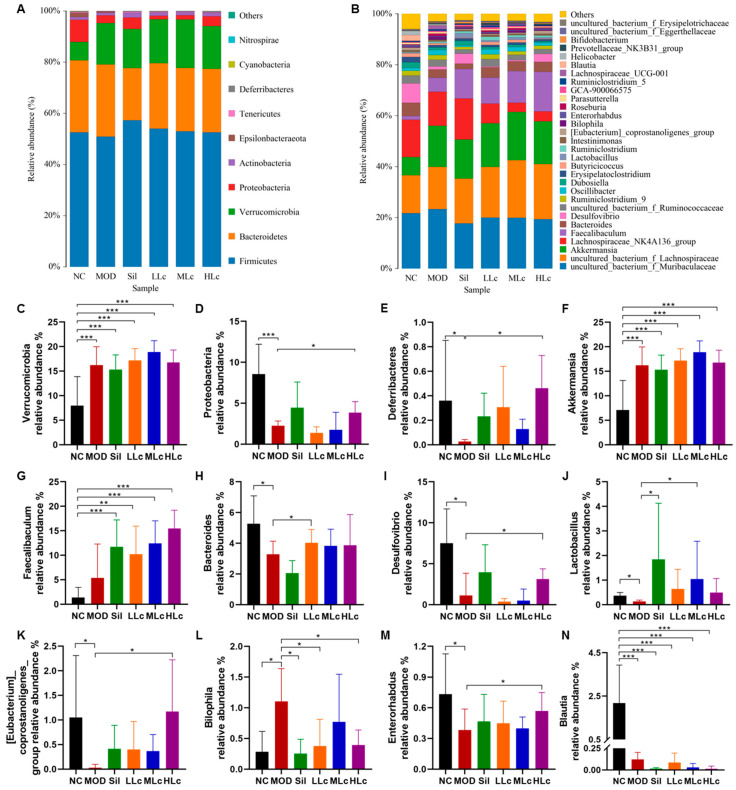
*Lactobacillus casei* modulates microbial taxonomic profiles in mice with anti-tuberculosis drugs: (**A**) relative abundance of the gut microbiota at the phylum level; (**B**) relative abundance of the gut microbiota at the genus level; (**C**) relative abundance of the Verrucomicrobia phylum; (**D**) relative abundance of the Proteobacteria phylum; (**E**) relative abundance of the Deferribacteres phylum; (**F**) relative abundance of the *Akkermansia* genus; (**G**) relative abundance of the *Faecalibaculum* genus; (**H**) relative abundance of the *Bacteroides* genus; (**I**) relative abundance of the *Desulfovibrio* genus; (**J**) relative abundance of the *Lactobacillus* genus; (**K**) relative abundance of the *[Eubacterium]_coprostanoligenes_group* genus; (**L**) relative abundance of the *Bilophila* genus; (**M**) relative abundance of the *Enterorhabdus* genus; (**N**) relative abundance of the *Blautia* genus. NC, normal control group; MOD, model group; Sil, positive control (silibinin) group; LLc, low-dose *Lactobacillus casei* group; MLC, medium-dose *Lactobacillus casei* group; HLC, high-dose *Lactobacillus casei* group. N = 8; * *p* < 0.05, ** *p* < 0.01, *** *p* < 0.001.

Changes in microbial taxonomic profiles were determined from the phylum to genus levels, and the gut microbiota compositions differed among the groups (Figure 5). The abundance of Proteobacteria and Deferribacteres was lower in the MOD group than in the NC group (*p* < 0.05), while the Verrucomicrobia abundance increased (Figure 5C–E). Conversely, high Lc significantly enhanced the Proteobacteria and Deferribacteres abundances, but it did not change the abundance of Verrucomicrobia. This result indicates that the changes in the abundance of Verrucobacteria were more likely caused by anti-tuberculosis drugs, while *Lactobacillus casei* altered the abundance of Proteobacteria and Deferribacteres. At the genus level, *Bacteroides*, *Desulfovibrio*, *Lactobacillus*, *[Eubacterium]_coprostanoligenes_group*, and *Enterorhabdus* displayed lower abundance in the MOD group than in the NC group (*p* < 0.05). In contrast to the MOD group, at least one of the Sil and LC-treated groups exhibited opposite variations in these bacteria, comparable to the control group’s levels (Figure 5H–K,M). In addition, the abundance of *Bilophila* bacteria was opposite from that previously described (Figure 5L). Furthermore, *Faecalibaculum* and *Akkermansia* were altered and enriched in other groups compared to the NC group, and *Blautia* abundance decreased in other groups (Figure 5F,G,N).

### 2.5. Lactobacillus casei Mediated Regulation of Intestinal Inflammation though the TLR4–NF-κB–MyD88 Pathway

**Figure 6:**
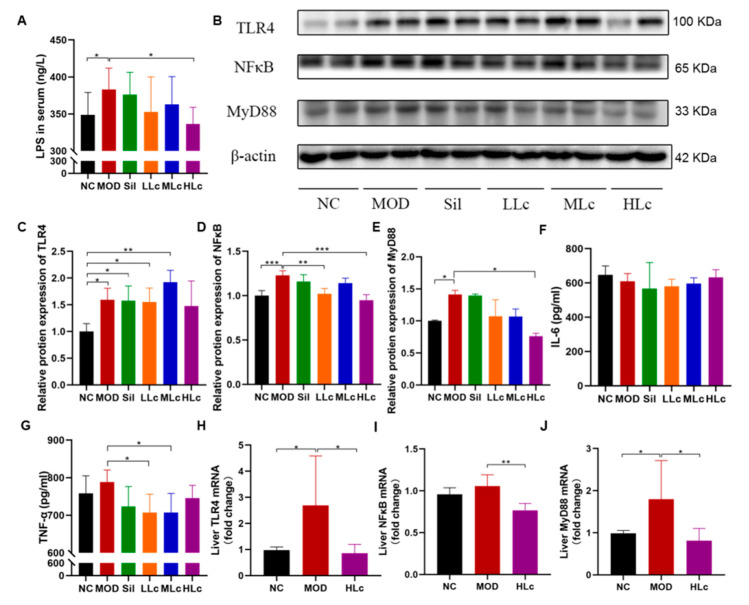
*Lactobacillus casei* mediated the regulation of intestinal inflammation though toll like receptor 4 (TLR4)–nuclear factor (NF)-κB–myeloiddifferentiationfactor 88 (MyD88) pathway: (**A**) serum lipopolysaccharide (LPS) level; (**B**) protein expression of TLR4, NFκB, and MyD88 measured by Western blot; (**C**) quantitative analysis of TLR4 protein expression; (**D**) quantitative analysis of NF-κB protein expression; (**E**) quantitative analysis of MyD88 protein expression; (**F**) liver interleukin (IL)-6 level; (**G**) liver tumor necrosis factor (TNF)-α level; (**H**) quantitative analysis of *TLR4* mRNA expression; (**I**) quantitative analysis of *NF-κB* mRNA expression; (**J**) quantitative analysis of *MyD88* mRNA expression. NC, normal control group; MOD, model group; Sil, positive control (silibinin) group; LLc, low-dose *Lactobacillus casei* group; MLC, medium-dose *Lactobacillus casei* group; HLC, high-dose *Lactobacillus casei* group. N = 9 for LPS, N = 3 for protein expression, N = 6 for IL-6, TNF-α, and mRNA expression; * *p* < 0.05, ** *p* < 0.01, *** *p* < 0.001.

As shown in Figure 6A, LPS content in the serum increased considerably in the MOD group compared to the NC group (*p* < 0.05). On the other hand, it was significantly lower in the HLc group than in the model group. Figure 6B–E illustrate the expressions of pathway proteins. The HLc group downregulated the expression of NF-κB and MyD88, which were upregulated by anti-tuberculosis drugs (Figure 6D,E). For another key pathway protein, TLR4 expression was increased by anti-TB drugs (*p* < 0.05, Figure 6C), and there was no statistical significance between the NC and HLc groups. Moreover, interleukin (IL)-6 and tumor necrosis factor (TNF)-α levels in the liver were measured to observe the hepatic inflammatory response (Figure 6F,G). There was no significant difference in the content of inflammatory factors among the groups; only after *Lactobacillus casei* intervention was the level of TNF-α significantly lower than that of the model group (Figure 6F,J). The mRNA expressions of *TLR4* and *MyD88* measured by quantitative real time polymerase chain reaction (qRT-PCR) were higher in the MOD than in the NC group (Figure 6H,J). Furthermore, *TLR4*, *NF-κB* and *MyD88* mRNA expressions were decreased in the HLc-treated group compared with the MOD group (Figure 6H–J).

### 2.6. Gut Microbiota-Associated Indicators of Hepatotoxicity, Degree of LPS, and Protein Expression Concentration

**Figure 7:**
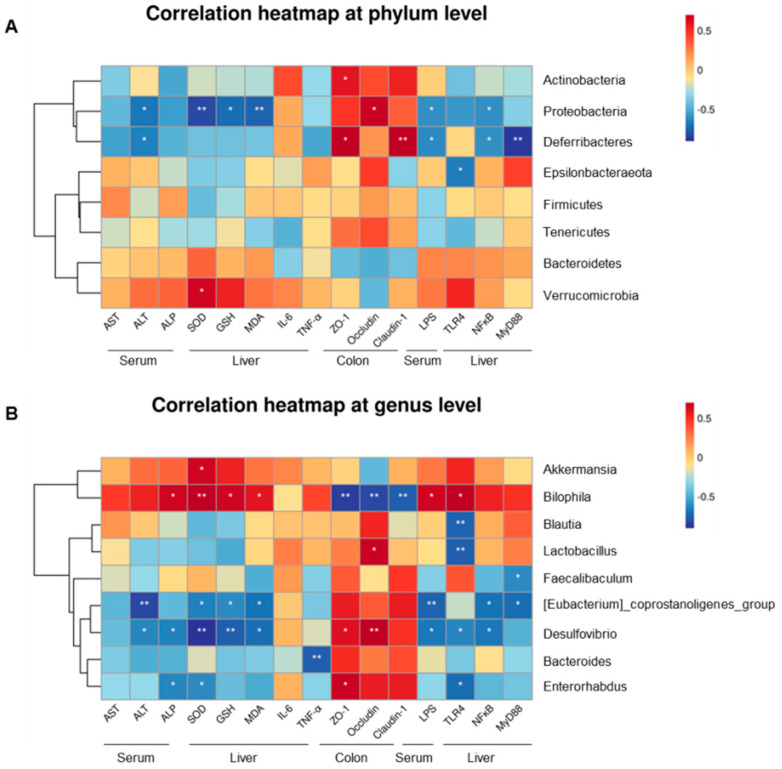
Relationship between the gut microflora and blood parameters, oxidative stress, inflammatory factors, level of LPS, and protein expression across groups (NC, MOD, and HLc), estimated by Spearman’s correlation analysis: (**A**) the seven phyla; (**B**) the nine genera. NC, normal control group; MOD, model group; HLC, high-dose *Lactobacillus casei* group. N = 4; * *p* < 0.05, ** *p* < 0.01.

At the phylum level, five phyla were significantly associated with serum transaminase, degree of LPS, oxidative stress, inflammatory cytokines, and colon and liver protein expression (Figure 7A). Deferribacteres and Proteobacteria were negatively correlated with serum ALT and LPS, respectively (*p* < 0.05). Meanwhile, oxidative stress factors were negatively correlated with the abundance of Proteobacteria. Actinobacteria, Deferribacteres, and Proteobacteria were positively correlated with ZO-1, occludin, and claudin-1 protein expression (*p* < 0.05). Figure 7B also depicts the correlation between the above indicators and gut microbiota at the genus level. *[Eubacterium]_coprostanoligenes_group* and *Desulfovibrio* revealed a negative connection with levels of ALT, oxidative stress factors, and NF-κB protein. *Bilophila* was negatively correlated with TJ proteins and had positive relationships with the concentrations of ALP, SOD, GSH, MDA, and TLR4. In addition, *Lactobacillus*, *Desulfovibrio*, and *Enterorhabdus* had positive associations with some tight junction proteins and negative associations with one or two pathway-relevant proteins. For LPS, *Bilophila* showed a positive correlation, whereas *[Eubacterium]_coprostanoligenes_group* and *Desulfovibrio* showed negative correlations.

## 3. Discussion

Anti-TB drug-induced liver injury is not conducive to the treatment and prognosis of tuberculosis patients. *Lactobacillus casei* plays an essential role in the adjuvant treatment of some liver diseases. In this study, *Lactobacillus casei* attenuated oxidative stress in the liver, repaired the intestinal barrier, regulated intestinal flora, alleviated LPS release into the blood, and modulated the TLR4–NF-κB–MyD88 pathway to attenuate liver injury caused by anti-tuberculosis drugs.

According to the 2019 Chinese Guidelines for the Diagnosis and Treatment of Anti-tuberculosis Drug-Induced Liver Injury [26], the diagnostic criteria for anti-tuberculosis drug-induced liver injury are as follows: after the use of anti-tuberculosis drugs, serum ALT no less than three times the upper limit of normal (ULN) and/or total bilirubin (TBIL) no less than two times the ULN; simultaneous increase in aspartate aminotransferase (AST), ALP, and TBIL, with at least one of them being no less than two times the ULN. Moreover, in a previous study, tuberculosis patients with ATDILI were not diagnosed with a significant increase in serum ALT and AST levels, consistent with our results on AST [27]. In this study, the elevation of ALP could also determine the occurrence of ATDILI. This is because ALP could be improved by *Lactobacillus casei* Zhang, indicating its positive significance in improving ATDILI. The improved effect of Lc on the liver injury was also reflected in the reduction in liver steatosis, inflammatory cell infiltration, and other pathological injuries. Interestingly, pretreatment with LcZ protected against LPS/d-galactosamine-induced liver damage in rats via its antioxidative and anti-inflammatory capacities [23,25]. Zhong et al. found that ATO + Sb cotreatment significantly increased SOD and SOD-1, as well as promoted the generation of MDA in liver tissues [28]. Chen’s study also showed TiO_2_ nanoparticles caused the accumulation of MDA and increased activity of glutathione peroxidase (GSH-Px) and SOD [29]. When it was first discovered, SOD was not associated with any negative effects. Later, studies showed that protective SOD concentrations might be restricted to a relatively narrow window in mammalian cells. SOD is very protective up to a point, beyond which it will promote lipid peroxidation, where its protection is lost, and injury is even exacerbated [30]. In this study, anti-TB drugs induced the increase in SOD, GSH, and MDA levels in the liver, thus resulting in oxidative stress. After the intervention of LcZ, the indicators improved, especially in the high-dose Lc group. On the other hand, a previous study indicated that the nuclear factor erythroid-2-related factor 2 (Nrf2) SOD anti-oxidant response element (ARE) signaling pathway is related not only to the endogenous antioxidant stress system but also to the detoxification regulatory conduction system in the body. Antioxidant enzymes, including SOD, can induce the transcription of cellular detoxification genes such as NADPH: Quinone Oxidoreductase 1(*NQO1*) [31]. This also provides a clue explaining why *Lactobacillus casei* improves ATDILI. In addition, compared with the model group, the Lc group decreased the level of TNF-α in the liver. This suggests that Lc suppressed ATDILI at least partly by reducing hepatic oxidative stress and the inflammatory response. However, silibinin’s preventive impact in this study was only shown in the pathological alterations of the liver. The additional intestinal protective effect of Lc could explain why silibinin was less effective in protecting ATDILI than Lc in this study [23].

Due to the gut–liver axis, the intestinal barrier and gut microbiota play important roles in liver diseases. Anti-TB drugs damaged the intestinal mucosa, induced severe inflammatory cell infiltration in colon tissue, and destroyed tight junctions. Notably, Lc intervention reversed inflammatory responses in the colon and upregulated tight junction protein expression. The results further demonstrated that Lc could alleviate intestinal inflammation by enhancing the intestinal barrier, as evidenced by a previous rat study [32]. In this study, anti-tuberculosis drugs reduced the Chao 1 index in mice, but Lc could not recover it. Interestingly, anti-tuberculosis drugs and Lc did not change the Shannon index, but the Shannon index of silibinin group mice was significantly lower than that of other groups. Ren and Shen’s studies indicated that silibinin could reduce or tend to reduce the diversity of intestinal flora [33,34], which may be related to the reduction in or even extinction of certain bacterial abundance by silibinin. For example, *Butyricicoccus* was an almost undetectable bacterium in the Sil group in this study, and its abundance was also proven to be reduced by silibinin in a previous study [34]. 

In addition, *Lactobacillus casei* also played a vital role in regulating GM. The abundance of Proteobacteria and Deferribacteres increased after Lc intervention compared with the MOD group. From the results, it is clear that anti-TB drugs and Lc could hardly alter the abundance of Firmicutes and Bacteroidetes, acting on Proteobacteria and Deferribacteres. Feng’s study proved that intestinal permeability was negatively correlated with Deferribacteres [35], corroborating our finding that Deferribacteres was positively associated with TJ proteins. From this, we speculate that the intake of higher doses of Lc may be more helpful in strengthening the intestinal barrier by increasing the abundance of Deferribacteres. Moreover, the abundance of Verrucomicrobia increased significantly after using anti-tuberculosis drugs. This increase in abundance appears to be closely related to *Akkermansia*. *Akkermansia* is a representative genus of Verrucomicrobia, and its intergroup abundance variation in this study was almost consistent with that of Verrucomicrobia. In both animals and humans, *Akkermansia* was found to bloom after exposure to specific antibiotics [36,37], which is consistent with our result, suggesting that *Akkermansia* may have resistance to anti-tuberculosis drugs. On the other hand, it is worth noting that Lc reversed many of the changes of GM caused by anti-tuberculosis drugs at the genus level, including *Lactobacillus*, *Bacteroides*, *Desulfovibrio*, and *Bilophila*. For example, *Bilophila* with a high-fat diet can promote higher inflammation and intestinal barrier dysfunction, leading to hepatic steatosis [38]. For *Lactobacillus*, its abundance did not represent the dose–effect relationship under different Lc intake amounts. In addition to the influence of individual differences, we know that *Lactobacillus* is a large genus of bacteria, and the increase of *Lactobacillus casei* content alone may not play a decisive role in the overall content. In future studies, we will also pay more attention to the changes in the intervenor-related bacteria genus. These changes in bacteria abundance indicate that Lc has a solid ability to regulate intestinal flora, explaining why it can alleviate intestinal and liver damage in mice. It has been reported that *Lactobacillus casei* can restore intestinal microecology damaged by antibiotics [24]. 

INH and RFP caused damage to the intestinal barrier in mice in our study, which was consistent with the results of LPS content in serum. LPS is the primary pathogen-associated molecular pattern and a natural TLR ligand [39]. Some authors have reported that the activation of TLR4 is involved in the development and progression of liver disease [13,40]. The difference in serum LPS levels among the groups revealed that Lc intervention, especially a high dosage of Lc, decreased the concentration of LPS in the blood increased by anti-tuberculosis drugs. In high-fat diet-fed mice, LPS induced gut–liver axis alteration through TLR4 overexpression and subsequent NF-κB signaling pathway activation, leading to a proinflammatory response mediated by TNF-α and IL-6 [40]. However, the changes in TNF-α and IL-6 in this study were atypical, revealing that anti-TB drugs only tended to increase TNF-α, whereas Lc reduced the TNF-α level. This difference might be attributed to the usage of anti-TB drugs, which are antibiotics in nature and have anti-inflammatory properties that may prevent inflammation from developing. Nonetheless, the present results prove that the TLR4–NF-κB–MyD88 pathway can be activated by anti-TB drugs, indicating that pathway activation plays an essential role in the gut–liver axis associated with ATDILI development. *Lactobacillus casei* upregulated the expression of intestinal TJ proteins, increased the abundance of beneficial bacteria such as Deferribacteres, *Desulfovibrio*, and *[Eubacterium]_coprostanoligenes_group*, and decreased the abundance of harmful bacteria such as *Bilophila*, potentially directly or indirectly enhancing the intestinal barrier. Because of this, the repaired intestinal barrier inhibited LPS release and, thus, failed to meet the conditions for pathway activation. As shown in this study, Lc inhibited the expression of NF-κB and MyD88, implying that Lc might suppress the liver’s TLR4–NF-κB–MyD88 signaling pathway for liver protection. Moreover, Lc did not inhibit the protein expression of TLR4, suggesting that Lc may also protect the liver by affecting other pathways, which requires further study.

The correlation analysis between gut microflora and other indices highlighted a close relationship between GM and liver injury. At the phylum level, Deferribacteres and Proteobacteria showed a positive correlation with TJ proteins but a negative correlation with signaling pathway proteins, demonstrating a protective effect. Therefore, increases in their abundance might contribute to the treatment of ATDILI. Moreover, as common Gram-negative bacteria, Proteobacteria’s abundance in this study was negatively correlated with serum LPS level, which may be because the production of LPS in the model group was not mainly dependent on Proteobacteria. The results also suggest that multiple factors could influence the correlation between the two. At the genus level, with the increase in abundance of the beneficial bacteria *Bacteroides*, *Lactobacillus*, *Blautia*, *Desulfovibrio*, and *Enterorhabdus*, the protein expression of TJ was significantly increased and/or the expression of signaling pathway proteins was significantly decreased. In particular, *Desulfovibrio* showed a stronger correlation. It was proven to be a powerful acetic acid generator and to alleviate nonalcoholic fatty liver disease in mice [20]. Our study considered that *Desulfovibrio* also played an important role in restoring anti-TB drug-induced liver injury. *Desulfovibrio* promotes the production of acetic acid to increase the content of short-chain fatty acids, which has been shown to work well in liver disease. In addition to acetic acid, *Desulfovibrio* is also a producer of H_2_S. For liver damage or chronic liver illnesses such as nonalcoholic fatty liver disease, a supplement of exogenous H_2_S in the form of NaHS was helpful [41]. In addition, *[Eubacterium]_coprostanoligenes_group* and *Desulfovibrio* were the only bacteria that showed significant negative correlations with ALT and LPS levels, suggesting that *[Eubacterium]_coprostanoligenes_group* has a similar probiotic effect to *Desulfovibrio*, and has incomparable advantages in liver protection. In addition, *Bilophila* showed a completely opposite correlation to the other bacteria mentioned above, indicating a potential hazard in ATDILI. It was demonstrated that these bacteria play important roles in the development of or recovery from ATDILI.

Our study was one of the few studies that investigated the protective effect and mechanism of probiotics against liver injury induced by anti-TB drugs. Meanwhile, this study compared the intervention effects of different doses of *Lactobacillus casei* and compared these effects with commonly used hepatoprotective drug, which has been rarely studied in previous research. In addition, we analyzed the correlation between GM and indicators of liver injury, providing new evidence for the study of the gut–liver axis in ATDILI. ATDILI is a complex pathological process involving many mechanisms. However, we only focused on the possible role of the TLR4–NF-κB–MyD88 pathway in this study. The role of GM and the gut–liver axis in ATDILI requires further exploration and research. In addition, it was observed that the alleviating effect of probiotics alone against ATDILI was limited, suggesting that further studies can concentrate on the protective effect of multiplex probiotics or combined prebiotics. Moreover, our study only provided some evidence that Lc had hepatoprotective effects. Future research with the revised RUCAM is required to determine whether Lc harms the liver.

## 4. Materials and Methods

### 4.1. Reagents

Isoniazid and silibinin were purchased from Sigma (St. Louis, MO, USA), and rifampicin was obtained from Tokyo Chemical Industries (Tokyo, Japan). The probiotic strain, *Lactobacillus casei* Zhang, was provided by the Key Laboratory of Dairy Biotechnology and Engineering, Ministry of Education, Inner Mongolia Agricultural University, China.

### 4.2. Animal and Ethics Statement

In total, 54 specific-pathogen-free male C57BL6/J mice (18–22 g in weight) were used (SiPeiFu Laboratory Animal Technology Co., Ltd., Beijing, China). The mice were housed in cages in an air-conditioned room (22 °C ± 1 °C) maintained at a constant relative humidity (50−60%) under a 12 h light–dark cycle. Mice received free access to drinking water (replacement at 3 day intervals) and food (commercial standard rodent chow: 18.0% protein, 4.0% lipids, and 5.0% fiber) during experiments. The animal procedures were carried out by the Guide for the Care and Use of Laboratory Animals and approved by the Qingdao University Laboratory Animal Welfare Ethics Committee (No.20210310C575420210514035).

### 4.3. Experimental Design

Mice were distributed into six groups (nine mice per group) according to the following treatments: NC, control group, mice received 0.9% normal saline, and then 0.5% sodium carboxymethyl cellulose 2 h later; MOD, model group, mice were administered 0.9% normal saline, followed by the combination of INH (150 mg/kg) and RFP (300 mg/kg) 2 h later; Sil, silibinin (positive control) group, mice were given 100 mg/kg silibinin, and then INH (150 mg/kg) and RFP (300 mg/kg) 2 h later; LLc (low-dose Lc group), MLc (medium-dose Lc group), and HLc (high-dose Lc group), mice were administered 1 × 10^8^ colony-forming unit (CFU), 1 × 10^9^ CFU, and 5 × 10^9^ CFU *Lactobacillus casei* per mouse, followed by the combination of INH and RFP in equal doses. All animals were treated by intragastric administration (0.2 mL/10 g per day) for 56 consecutive days. INH and RFP were dissolved in 0.5% sodium carboxymethyl cellulose (CMC). CMC is a good solvent and is not toxic at the right dose; it has been used as a thickener or carrier for medicine [42]. In a previous study, CMC was used as a solvent for anti-tuberculosis drugs in animal experiments [43]. Body weight and food intake were monitored weekly. Animals were sacrificed the morning following the last administration. We collected and weighed serum, liver, and colon tissue samples. The cecum and its contents were stored at –80 °C for further determination.

### 4.4. Hematological Tests of Hepatic Function and Inflammatory Cytokines

We tested the levels of liver function indicators in serum, including ALT, AST, and ALP, using a fully automatic biochemical analyzer. Levels of SOD, GSH, and MDA in the liver were detected using kits (Nanjing Jiancheng Biological Engineering Institute, Nanjing, China). Levels of interleukin-6 (IL-6) and tumor necrosis factor-α (TNF-α) in the liver were measured by ELISA kits (ABclonal, Wuhan, China).

### 4.5. Serum LPS Level

The level of serum LPS was assessed using an ELISA kit from Jingkang Biotechnology Co., Ltd. (Shanghai, China).

### 4.6. Histological Examination

Fresh liver and colon tissues were collected and soaked in 4% paraformaldehyde. After the alcohol was dehydrated, the tissues were embedded (in paraffin), sliced, and stained with hematoxylin and eosin. Images of the tissue sections were acquired using an Olympus corporation microscope (Tokyo, Japan). After observing the pictures, five visual fields were randomly selected for each group of mice, and the histological activity index score for the liver [44] and histological colitis score for the colon [45] were determined. The HAI score is the combined scores for necrosis, inflammation, and fibrosis of the liver, and the scoring criteria are shown in Table 1. The histological colitis score is shown in Table 2.

### 4.7. 16S rRNA Amplicon Sequencing Analyses

The main composition of the microbiomes in the cecal contents were analyzed by qPCR (n = 8). Cecal contents were collected aseptically and sent to Biomarker Biotechnology Co., Ltd. (Beijing, China). The extraction of genomic DNA from cecal samples was carried out using the QIAamp Fast DNA stool Mini Kit (Qiagen, DEU, Hilden, Germany) according to the manufacturer’s instructions. After the quality was verified, PCR amplification was conducted with barcoded specific bacterial primers targeting the variable region 3–4 (V3–V4) of the 16S rRNA gene: forward primer 338F, 5′–ACTCCTACGGGAGGCAGCA–3′; reverse primer 806R, 5′–GGACTACHVGGGTWTCTAAT–3′. Relative analysis was performed using BMKCloud (www.biocloud.net, accessed on 15 May 2023), including the number of OTUs, α diversity (Chao1 index and Shannon index), β diversity, and microbial taxonomic profile analysis. The specific analysis methods were conducted as previously described [32].

### 4.8. Western Blot

The expression of the tight junction (TJ) proteins, including ZO-1, occludin, and claudin-1, in the colon and of pathway proteins, such as TLR4, NF-κB, and MyD88, in the liver were detected by Western blot. Colon and liver tissues (0.05 g) were lysed on ice with 500 μL of RIPA lysis buffer (Beyotime, Shanghai, China) for 30 min in the presence of protease and phosphatase inhibitors. After centrifugation at 12,000 rpm for 4 min at 4 °C, the supernatant was harvested. Then, protein concentrations were determined using the BCA protein assay kit (BOSTER, Wuhan, China). and the protein was thermally denatured at 100 °C for 10 min. Equivalent amounts of protein from each sample were separated by 8% or 10% sodium dodecyl sulfate (SDS)-polyacrylamide gel electrophoresis (PAGE) and then electrophoretically transferred onto polyvinylidene fluoride membranes. After blocking with 5% fat-free milk in tris buffered saline with tween (TBST) buffer, the membranes were incubated with the corresponding primary antibodies overnight at 4 °C: mouse anti-mouse primary antibody β-actin (BOSTER, China) and rabbit anti-mouse primary antibodies, including ZO-1(Novus, Centennial, CO, USA), occludin (Abcam, Cambridge, MA, USA), claudin-1 (Novus, USA), TLR4 (Abclone, Wuhan, China), NF-κB (CST, Boston, MA, USA), and MyD88 (CST, USA). Then, the membranes were incubated with HRP-conjugated secondary antibodies. The membranes were treated with ECL photoluminescence solution for imaging. Protein band densities were analyzed using ImageJ software (Version 1.8.0).

### 4.9. Real-Time Quantitative PCR (qRT-PCR)

The total RNA of each sample was extracted from the liver according to the instruction manual of the Trizol Reagent (Beyotime, Nantong, China). The gene sequences of *TLR4*, *NF-κB*, and *MyD88* in mice were searched on NCBI, and primers were synthesized by Accurate Biology. Table 3 shows the primer sequences. Reverse transcription and qRT-PCR were performed in strict accordance with the manufacturer’s instructions (Accurate Biology, Changsha, China). 

### 4.10. Statistical Analysis

GraphPad Prism 8.0 software was used for statistical analysis. and all experimental data were expressed as means ± standard deviation (SD). Student’s *t*-test and one-way ANOVAs were used to determine the significance of the difference. In addition, the LSD *t*-test was used to compare the statistical differences among multiple groups. Differences in β diversity were visualized using principal component analysis (PCA) and PCoA. Permutational multivariate analysis of variance (PERMANOVA) using the weighted UniFrac similarity coefficient based on binary Jaccard was also performed in PCoA. Spearman correlation analyses were used to assess the correlations among serum transaminase, protein expression, degree of LPS, inflammatory cytokines, and gut microbiota. A *p*-value <0.05 was considered significantly different.

## 5. Conclusions

INH and RFP can cause anti-tuberculosis drug-induced liver injury. Anti-tuberculosis drugs also impaired the intestinal barrier in mice, reduced GM diversity, and altered its composition. Lc strengthened the intestinal barrier and restored gut microflora to near-normal composition. Anti-tuberculosis drugs activated the TLR4–NF-κB–MyD88 pathway to induce liver injury, and high-dose Lc intervention inhibited the activation of NF-κB and MyD88 proteins, thus reducing oxidative stress and inflammatory response, as well as alleviating liver injury. Meanwhile, there were close relationships between GM and indicators related to liver injury and the intestinal barrier. These findings indicate that Lc could relieve liver injury and restore the gut state, which may be a potentially effective adjuvant treatment for ATDILI.

## Figures and Tables

**Table 1:** The scoring criteria of HAI.

Category		Scores and Criteria					
Periportal with or without bridging necrosis	0: none	1: mild piecemeal necrosis	3: moderate piecemeal necrosis (less than involvement around the portal tracts)	4: marked piecemeal necrosis (more than involvement around the portal tracts)	5: moderate piecemeal necrosis plus bridging necrosis	6: marked piecemeal necrosis plus bridging necrosis	10: multilobular necrosis
Intralobular degeneration and focal necrosis	0: none	1: mild (acidophilic bodies, ballooning degeneration and/or scattered foci of hepatocellular necrosis in <1/3 of lobules or nodules)	3: moderate (involvement of 1/3–2/3 of lobules or nodules)	4: marked (involvement of >2/3 of lobules or nodules)			
Portal inflammation	0: no portal inflammation	1: mild (sprinkling of inflammatory cells in <1/3 of portal tracts)	3: moderate (increased inflammatory cells in 1/3–2/3 of portal tracts)	4: marked (dense packing of inflammatory cells in >2/3 of portal tracts)			
Fibrosis	0: no fibrosis	1: fibrous portal expansion	3: bridging fibrosis (portal–portal or portal–central linkage)	4: cirrhosis			

**Table 2:** The scoring criteria of histological colitis score.

Category	Scores and Criteria				
Inflammation	0: none	1: slight	2: moderate	3: severe	
Extent	0: none	1: mucosa	2: mucosa and submucosa	3: transmural	
Regeneration	0: complete regeneration or normal tissue	1: almost complete regeneration	2: regeneration with crypt depletion	3: surface epithelium not intact	4: no tissue repair
Crypt damage	0: none	1: basal 1/3 damaged	2: basal 2/3 damaged	3: only surface epithelium	4: entire crypt and epithelium lost
Percent involvement	1: 1–25%	2: 26–50%	3: 51–75%	4: 76–100%	

**Table 3:** Primer sequences.

Name	Sequence (5′–3′)
TLR4	F: 5′–ACACCTACCTGGAATGGGAGG–3′ R: 5′–TCAGGTCCAAGTTGCCGTTTC–3′
NF-κB	F: 5′–CCATTGTGTTCCGGACTCCTC–3′ R: 5′–GTGGCGATCATCTGTGTCTGG–3′
MyD88	F: 5′–TACAGGTGGCCAGAGTGGAA–3′ R: 5′–GCAGTAGCAGATAAAGGCATCGAA–3′

## Data Availability

No new data were created or analyzed in this study. Data sharing is not applicable to this article.

## References

[1] Furin J., Cox H., Pai M. (2019). Tuberculosis. Lancet.

[2] Yew W.W., Chang K.C., Chan D.P. (2018). Oxidative Stress and First-Line Antituberculosis Drug-Induced Hepatotoxicity. Antimicrob. Agents Chemother..

[3] Cavaco M.J., Alcobia C., Oliveiros B., Mesquita L.A., Carvalho A., Matos F., Carvalho J.M., Villar M., Duarte R., Mendes J. (2022). Clinical and Genetic Risk Factors for Drug-Induced Liver Injury Associated with Anti-Tuberculosis Treatment—A Study from Patients of Portuguese Health Centers. J. Pers. Med..

[4] Low E.X.S., Zheng Q., Chan E., Lim S.G. (2020). Drug induced liver injury: East versus West—A systematic review and meta-analysis. Clin. Mol. Hepatol..

[5] Xu Z.W., Li Y., Xu J.M. (2013). Clinical characteristics of the adaptive phenomenon of antituberculosis drug-induced liver injury. Chin. J. Hepatol..

[6] Zhang S., Pan H., Peng X., Lu H., Fan H., Zheng X., Xu G., Wang M., Wang J. (2016). Preventive use of a hepatoprotectant against anti-tuberculosis drug-induced liver injury: A randomized controlled trial. J. Gastroenterol. Hepatol..

[7] Di Costanzo A., Angelico R. (2019). Formulation Strategies for Enhancing the Bioavailability of Silymarin: The State of the Art. Molecules.

[8] Stickel F., Schuppan D. (2007). Herbal medicine in the treatment of liver diseases. Dig. Liver Dis. Off. J. Ital. Soc. Gastroenterol. Ital. Assoc. Study Liver.

[9] Zhang J., Zhao Z., Bai H., Wang M., Jiao L., Peng W., Wu T., Liu T., Chen H., Song X. (2019). Genetic polymorphisms in PXR and NF-κB1 influence susceptibility to anti-tuberculosis drug-induced liver injury. PLoS ONE.

[10] Schneider K.M., Bieghs V., Heymann F., Hu W., Dreymueller D., Liao L., Frissen M., Ludwig A., Gassler N., Pabst O. (2015). CX3CR1 is a gatekeeper for intestinal barrier integrity in mice: Limiting steatohepatitis by maintaining intestinal homeostasis. Hepatology.

[11] Yan A.W., Fouts D.E., Brandl J., Stärkel P., Torralba M., Schott E., Tsukamoto H., Nelson K.E., Brenner D.A., Schnabl B. (2011). Enteric dysbiosis associated with a mouse model of alcoholic liver disease. Hepatology.

[12] Hua Q., Han Y., Zhao H., Zhang H., Yan B., Pei S., He X., Li Y., Meng X., Chen L. (2022). Punicalagin alleviates renal injury via the gut-kidney axis in high-fat diet-induced diabetic mice. Food Funct..

[13] Liu Q., Tian H., Kang Y., Tian Y., Li L., Kang X., Yang H., Wang Y., Tian J., Zhang F. (2021). Probiotics alleviate autoimmune hepatitis in mice through modulation of gut microbiota and intestinal permeability. J. Nutr. Biochem..

[14] Liu S., Chen Q., Liu J., Yang X., Zhang Y., Huang F. (2018). Sinomenine protects against *E. coli*-induced acute lung injury in mice through Nrf2-NF-κB pathway. Biomed. Pharmacother..

[15] Tang J., Xu L., Zeng Y., Gong F. (2021). Effect of gut microbiota on LPS-induced acute lung injury by regulating the TLR4/NF-kB signaling pathway. Int. Immunopharmacol..

[16] Ju M., Liu B., He H., Gu Z., Liu Y., Su Y., Zhu D., Cang J., Luo Z. (2018). MicroRNA-27a alleviates LPS-induced acute lung injury in mice via inhibiting inflammation and apoptosis through modulating TLR4/MyD88/NF-κB pathway. Cell Cycle.

[17] Hu N., Wang C., Dai X., Zhou M., Gong L., Yu L., Peng C., Li Y. (2020). Phillygenin inhibits LPS-induced activation and inflammation of LX2 cells by TLR4/MyD88/NF-κB signaling pathway. J. Ethnopharmacol..

[18] Hayden M.S., Ghosh S. (2011). NF-κB in immunobiology. Cell Res..

[19] Raftar S.K.A., Ashrafian F., Abdollahiyan S., Yadegar A., Moradi H.R., Masoumi M., Vaziri F., Moshiri A., Siadat S.D., Zali M.R. (2022). The anti-inflammatory effects of *Akkermansia muciniphila* and its derivates in HFD/CCL4-induced murine model of liver injury. Sci. Rep..

[20] Hong Y., Sheng L., Zhong J., Tao X., Zhu W., Ma J., Yan J., Zhao A., Zheng X., Wu G. (2021). Desulfovibrio vulgaris, a potent acetic acid-producing bacterium, attenuates nonalcoholic fatty liver disease in mice. Gut Microbes.

[21] Yan R., Wang K., Wang Q., Jiang H., Lu Y., Chen X., Zhang H., Su X., Du Y., Chen L. (2022). Probiotic *Lactobacillus casei* Shirota prevents acute liver injury by reshaping the gut microbiota to alleviate excessive inflammation and metabolic disorders. Microb. Biotechnol..

[22] Zhu H., Cao C., Wu Z., Zhang H., Sun Z., Wang M., Xu H., Zhao Z., Wang Y., Pei G. (2021). The probiotic *L. casei* Zhang slows the progression of acute and chronic kidney disease. Cell Metab..

[23] Wang Y., Xie J., Li Y., Dong S., Liu H., Chen J., Wang Y., Zhao S., Zhang Y., Zhang H. (2016). Probiotic *Lactobacillus casei* Zhang reduces pro-inflammatory cytokine production and hepatic inflammation in a rat model of acute liver failure. Eur. J. Nutr..

[24] Yao G., Cao C., Zhang M., Kwok L.Y., Zhang H., Zhang W. (2021). *Lactobacillus casei* Zhang exerts probiotic effects to antibiotic-treated rats. Comput. Struct. Biotechnol. J..

[25] Wang Y., Li Y., Xie J., Zhang Y., Wang J., Sun X., Zhang H. (2013). Protective effects of probiotic *Lactobacillus casei* Zhang against endotoxin- and d-galactosamine-induced liver injury in rats via anti-oxidative and anti-inflammatory capacities. Int. Immunopharmacol..

[26] Society of Tuberculosis C.M.A. (2019). Guidelines for diagnosis and treatment of anti-tuberculosis drug-induced liver injury (2019 edition). Chin. J. Tuberc. Respir. Dis..

[27] Su Q., Liu Q., Liu J., Fu L., Liu T., Liang J., Peng H., Pan X. (2021). Study on the associations between liver damage and antituberculosis drug rifampicin and relative metabolic enzyme gene polymorphisms. Bioengineered.

[28] Zhong G., Wan F., Wu S., Jiang X., Tang Z., Zhang X., Huang R., Hu L. (2021). Arsenic or/and antimony induced mitophagy and apoptosis associated with metabolic abnormalities and oxidative stress in the liver of mice. Sci. Total Environ..

[29] Chen Z., Zhou D., Han S., Zhou S., Jia G. (2019). Hepatotoxicity and the role of the gut-liver axis in rats after oral administration of titanium dioxide nanoparticles. Part. Fibre Toxicol..

[30] McCord J.M., Edeas M.A. (2005). SOD, oxidative stress and human pathologies: A brief history and a future vision. Biomed. Pharmacother..

[31] Zhang Q., Zhang C., Ge J., Lv M.W., Talukder M., Guo K., Li Y.H., Li J.L. (2020). Ameliorative effects of resveratrol against cadmium-induced nephrotoxicity via modulating nuclear xenobiotic receptor response and PINK1/Parkin-mediated Mitophagy. Food Funct..

[32] Li Y., Zhao L., Hou M., Gao T., Sun J., Luo H., Wang F., Zhong F., Ma A., Cai J. (2022). *Lactobacillus casei* Improve Anti-Tuberculosis Drugs-Induced Intestinal Adverse Reactions in Rat by Modulating Gut Microbiota and Short-Chain Fatty Acids. Nutrients.

[33] Ren L., Ma X., Wang H., Li R., Cui J., Yan P., Wang Y., Yu X., Du P., Yu H. (2022). Prebiotic-like cyclodextrin assisted silybin on NAFLD through restoring liver and gut homeostasis. J. Control. Release Off. J. Control. Release Soc..

[34] Shen L., Liu L., Li X., Ji H. (2019). Regulation of gut microbiota in Alzheimer’s disease mice by silibinin and silymarin and their pharmacological implications. Appl. Microbiol. Biotechnol..

[35] Feng Y., Huang Y., Wang Y., Wang P., Song H., Wang F. (2019). Antibiotics induced intestinal tight junction barrier dysfunction is associated with microbiota dysbiosis, activated NLRP3 inflammasome and autophagy. PLoS ONE.

[36] Nobel Y.R., Cox L.M., Kirigin F.F., Bokulich N.A., Yamanishi S., Teitler I., Chung J., Sohn J., Barber C.M., Goldfarb D.S. (2015). Metabolic and metagenomic outcomes from early-life pulsed antibiotic treatment. Nat. Commun..

[37] Vrieze A., Out C., Fuentes S., Jonker L., Reuling I., Kootte R.S., van Nood E., Holleman F., Knaapen M., Romijn J.A. (2014). Impact of oral vancomycin on gut microbiota, bile acid metabolism, and insulin sensitivity. J. Hepatol..

[38] Natividad J.M., Lamas B., Pham H.P., Michel M.L., Rainteau D., Bridonneau C., da Costa G., van Hylckama Vlieg J., Sovran B., Chamignon C. (2018). Bilophila wadsworthia aggravates high fat diet induced metabolic dysfunctions in mice. Nat. Commun..

[39] Calabrese V., Cighetti R., Peri F. (2015). Molecular simplification of lipid A structure: TLR4-modulating cationic and anionic amphiphiles. Mol. Immunol..

[40] Porras D., Nistal E., Martínez-Flórez S., Pisonero-Vaquero S., Olcoz J.L., Jover R., González-Gallego J., García-Mediavilla M.V., Sánchez-Campos S. (2017). Protective effect of quercetin on high-fat diet-induced non-alcoholic fatty liver disease in mice is mediated by modulating intestinal microbiota imbalance and related gut-liver axis activation. Free Radic. Biol. Med..

[41] Sutti S., Locatelli I., Bruzzì S., Jindal A., Vacchiano M., Bozzola C., Albano E. (2015). CX3CR1-expressing inflammatory dendritic cells contribute to the progression of steatohepatitis. Clin. Sci..

[42] Layek B., Mandal S. (2020). Natural polysaccharides for controlled delivery of oral therapeutics: A recent update. Carbohydr. Polym..

[43] Liu X., Zhao M., Mi J., Chen H., Sheng L., Li Y. (2017). Protective Effect of Bicyclol on Anti-Tuberculosis Drug Induced Liver Injury in Rats. Molecules.

[44] Knodell R.G., Ishak K.G., Black W.C., Chen T.S., Craig R., Kaplowitz N., Kiernan T.W., Wollman J. (1981). Formulation and application of a numerical scoring system for assessing histological activity in asymptomatic chronic active hepatitis. Hepatology.

[45] Dieleman L.A., Palmen M.J., Akol H., Bloemena E., Peña A.S., Meuwissen S.G., Van Rees E.P. (1998). Chronic experimental colitis induced by dextran sulphate sodium (DSS) is characterized by Th1 and Th2 cytokines. Clin. Exp. Immunol..

